# Janus Kinase Inhibitors in Rheumatoid Arthritis: An Update on the Efficacy and Safety of Tofacitinib, Baricitinib and Upadacitinib

**DOI:** 10.3390/jcm12206690

**Published:** 2023-10-23

**Authors:** Robert Harrington, Patricia Harkins, Richard Conway

**Affiliations:** Department of Rheumatology, St. James’s Hospital, James Street, Dublin 8, D08 NHY1 Dublin, Ireland; harkinp@tcd.ie

**Keywords:** JAK inhibitor, JAKi, Janus kinase inhibitior, efficacy, safety, RA, rheumatoid arthritis, ORAL surveillance, MACE, thromboembolism, VTE, malignancy

## Abstract

Janus kinase inhibitors (JAKis) are the most recent new drug class to arrive to the market for rheumatoid arthritis (RA) treatment. While they have proven to be a very effective treatment option, there remains significant concern regarding the risk of cardiovascular events, thrombosis and malignancy, particularly given the findings of the post-marketing ORAL Surveillance study and FDA black box warnings. This article reviews the key findings of the most impactful cohort of studies and registry data since ORAL Surveillance. It also evaluates the role of JAKis in practice and offers guidance on risk stratifying patients and determining their suitability for a JAKi.

## 1. Introduction

Rheumatoid arthritis (RA) is a chronic systemic inflammatory disease characterised by joint pain, swelling and progressive damage. Without treatment RA can cause significant pain, deformity, loss of function and deterioration in overall quality of life [1]. Methotrexate (MTX) has been the first-line disease-modifying anti-rheumatic drug (DMARD) for RA since the late 1990s and it remains so today [2]. Despite dose escalation with MTX, not all patients will achieve disease remission or a low disease activity (LDA) state. Approximately 30% will discontinue MTX within the first year of commencing treatment due to either a lack of efficacy or intolerable side effects [3].

As a result, the last three decades have seen a continued drive to better understand and treat RA with the arrival of multiple biologic DMARDs (bDMARDs) to the market. The first tumour necrosis factor (TNF) inhibitor etanercept was introduced a quarter of a century ago in 1998. Other mechanisms of action (MOA) have been explored and bDMARDs today include MOAs that deplete B cells, modulate the T-cell pathway and inhibit interleukin 6 (IL-6). However there remains a sizeable cohort of RA patients with suboptimal control, loss of response or intolerability to the existing bDMARDs [1]. Even with the advances in bDMARDs, only 40–50% of patients achieve disease remission [4,5]. The need for a new therapeutic class drove the development of the novel oral janus kinase inhibitor (JAKi) class which has demonstrated impressive efficacy in terms of the American College of Rheumatology (ACR) response rates and disease activity scores (DAS28). Not only is there an impressive reduction in the number of swollen joints but there is a notable rapid reduction in pain scores and a robust arresting effect on long term radiographic progression. Nevertheless, the arrival of the JAKi class to market has been met with caution by the prescribing rheumatologists, given concerning safety signals with respect to major adverse cardiovascular events (MACE) and malignancy.

## 2. A Novel Mechanism of Action Brings New Concerns

**Figure 1:**
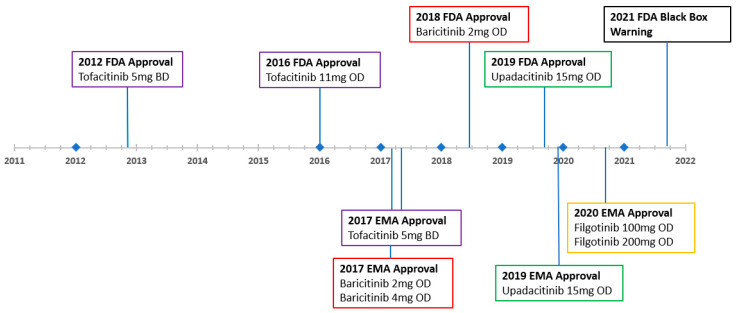
Timeline of JAK Inhibitor Approval [7].

The discovery of the role of JAK and the signal transducer and activator of transcription (STAT) constituents of cytokine signally in RA provided a new target for drug development. The arrival of the JAKi class was initially heralded for its perceived targeted “smarter” mechanism of action and its oral route of delivery. The new class was seen as an excellent option for a needle-phonic cohort of patients for which “home injections” and medication compliance was a challenging issue [1]. Tofacitinib (TOFA) was the first JAK inhibitor (JAKi) approved for RA patients in 2012. Four other JAKis have since been approved for RA; baricitinib (BARI), upadacitinib (UPA), filgotinib and peficitinib [6]. The timeline for the various JAKis approved by the Food and Drug Administration (FDA) and the European Medicines Agency (EMA) are shown in Figure 1.

Initial efficacy in terms of ACR response rates and DAS28 scores were very promising with both TOFA and BARI demonstrating comparative efficacy to the TNFi class. However, after the arrival of TOFA to market, a post-marketing safety surveillance study by the ORAL Surveillance trial was mandated by the FDA because of a possible concerning safety signal with regards to cardiovascular events and thrombotic events. Even prior to the ORAL Surveillance, concerning safety signals appeared during the trials of BARI regarding increased risk of venous thromboembolism (VTE) suggesting a possible class effect of JAK inhibitors, rather than a risk associated solely with TOFA and strong JAK1/JAK3 inhibition [8]. Before we focus on the potential negatives of the JAKi class, we must first look at the positives which have been somewhat overlooked since the furore caused by the FDA black box warnings.

## 3. Efficacy in RCTs

The JAKis approved by the FDA, namely tofacitinib (TOFA), baricitinib (BARI) and upadacitinib (UPA), have all demonstrated undoubted clinical efficacy in terms of ACR response rates, DAS28 scores and patient pain scores. It is generally accepted that, as a monotherapy, all JAKis are more effective than conventional DMARDs (cDMARDs) given the superiority demonstrated by TOFA, BARI and UPA versus MTX in RCTs [9,10,11,12]. Subsequent completion of four head-to-head RCTs with the TNFi comparator adalimumab (ADA), strongly suggests that the JAKi class is at least as effective, if not more so [13,14,15,16].

While there are no head-to-head trials for the available JAKis in RA, Lee at al. performed a network meta-analysis to compare the relative efficacy and safety profiles of TOFA, BARI, UPA, filgotinib and peficitinib as monotherapies for RA. Of the FDA approved JAKis, this meta-analysis suggested that BARI 4 mg + MTX and UPA 15 mg + MTX achieve the highest ACR response rates, with significantly higher ACR20 response rates compared to ADA 40 mg + MTX [17].

**Figure 2:**
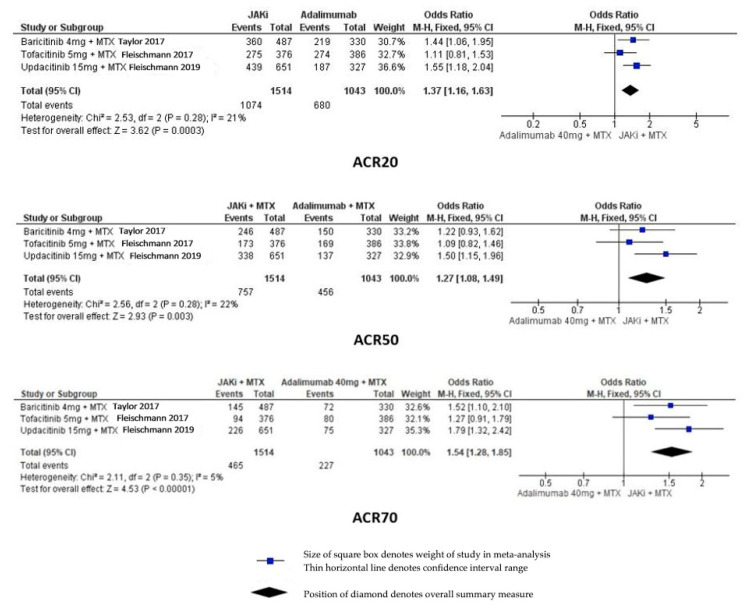
Forest Plots of ACR Scores for the 3 FDA Approved JAKis [13,15,16].

Pooling the data from the TOFA, BARI and UPA RCTs would seem to suggest that the combination of a JAKi with MTX is more efficacious than the combination of a TNFi with MTX in terms of ACR20, ACR50 and ACR70. Forest plots of relative efficacy of JAKis are shown in Figure 2.

## 4. Real World Evidence and Comparison to TNFi and Abatacept

The current evidence would also suggest that TOFA, BARI and UPA are efficacious in patients who have refractory RA resistant to previous bDMARD treatment [18,19,20]. Real world data from the Japanese FIRST registry also demonstrated the efficacy of JAKis in a cohort of RA patients with refractory disease despite two or more previous bDMARDs or targeted synthetic DMARDs. This Japanese cohort study of difficult to treat RA found that the JAKi class was associated with the highest proportion of rapid responders and the best outcomes in clinical disease activity [21]. While again reiterating that there are no head-to-head trials of JAKis, AbbVie’s Rinvoq (UPA) can claim superiority to bDMARDs such as the TNFi adalimumab (ADA) and the cytotoxic T-lymphocyte antigen-4 inhibitor (CTLA-4i) abatacept (ABA) based on the results of SELECT–COMPARE and SELECT–CHOICE, respectively [15,22].

## 5. Slowing of Structural Disease Progression

TOFA, BARI and UPA all have a solid evidence base in terms of slowing articular damage as assessed by the modified van der Heijde Total Sharp Score. TOFA monotherapy was shown to be superior to MTX monotherapy in limiting progression of structural damage in ORAL-START [9]. Both BARI and ADA fared favourably in terms of radiographic progression as early as week 24 when compared with MTX in RA-BEAM [13]. BARI + MTX was also statistically superior to MTX monotherapy in terms of the reduction in radiographic progression in RA-BEGIN [23]. Lastly, UPA performed similarly to ADA using the modified van der Heijde score, erosion score and joint space narrowing as metrics of radiographic progression in SELECT–COMPARE [15]. Notably the patients enrolled in this trial were all RA patients with an inadequate response to MTX. They were randomised to three groups: UPA + MTX, ADA + MTX and Placebo + MTX.

## 6. A Potential Novel Option in RA-ILD

Over the past few decades uncertainty has surrounded many csDMARDs and bDMARDs when it comes to their roles in lung involvement in RA. Much has been made of the association between MTX and RA interstitial lung disease (RA-ILD). Ultimately this was due to a historic channelling bias as patients with more severe RA were traditionally both more likely to develop RA-ILD and to be treated with MTX [24,25]. In fact the opposite is true, as the seminal ERAS and ERAN studies which recruited 2701 RA patients showed no association between MTX exposure and incident RA-ILD [26]. On the contrary, MTX exposure was associated with significantly less RA-ILD, and this would suggest a protective effect in delaying the onset of ILD [27]. Similar concerns were raised after the introduction of the TNFi class with early reports suggesting a worsening of underlying ILD [28]. Fortunately, subsequent studies allayed these concerns, as similar rates of ILD were seen across all classes of bDMARDs [29,30,31]. Nevertheless, the pursuit of further studies to determine the best drug class to treat RA-ILD is important. At the current time, aside from tight RA disease control with up-titration of MTX, the accepted limited evidence base would suggest that rituximab and abatacept are the best options to add to background MTX. More recently, small real world cohort studies suggest a similar beneficial effect of the JAKi class on RA-ILD stabilisation as ABA [32]. JAKis have also demonstrated a greater reduction in KL-6 levels than other bDMARDs and it is thought that this may indicate a better stabilising effect on RA-ILD disease activity [33].

The most compelling evidence on the potential beneficial role of the JAKi class on RA-ILD stabilisation comes from a recent large retrospective cohort study of over 28,000 RA patients in the United States. The patients treated with TOFA had the lowest incidence of ILD compared with patients treated with all bDMARDs. After adjusting for covariates, TOFA demonstrated a 69% reduced risk of ILD (adjusted hazard ratio 0.31; 95% CI, 0.12–0.78; *p* = 0.0090) compared with patients treated with ADA [34]. This suggested beneficial effect requires replication in further studies of RA-ILD patients.

While the young, active “needle phobic” patient and the multi-bDMARD disease resistant patient are two groups that would be benefit from JAKi treatment, perhaps the rarer RA-ILD patient is a third smaller subgroup where the risk-benefit analysis is in favour of opting for a JAKi.

## 7. MACE

After the arrival of TOFA to market, the FDA in the United States mandated a post-marketing surveillance study because of possible concerning cardiovascular safety signals seen in RA patients receiving JAKi treatment. ORAL Surveillance was a 4 year randomised, open-label, non-inferiority, safety end-point trial, in which patients of 50 years of age or older with active RA despite MTX treatment, and with at least one additional cardiovascular risk factor, were randomly assigned in a 1:1:1 ratio to receive TOFA at a dose of 5 mg or 10 mg twice daily or a subcutaneous TNF inhibitor, i.e., etanercept or adalimumab [35].

Significantly, non-inferiority criteria were not met for malignancies or major adverse cardiovascular events (MACE) [36]. After 4 years, TOFA compared less favourably to TNFi with hazard ratios of 1.33 (95% CI 0.91 to 1.94) for MACE and 1.48 (95% CI 1.04 to 2.09) for cancers. The authors estimated that during 5 years of treatment, 113 patients would have to be treated with TOFA as opposed to a TNFi to result in one additional MACE. They also estimated that 55 patients would have to be treated with TOFA as opposed to a TNFi to result in one additional malignancy over the same time frame.

**Figure 3:**
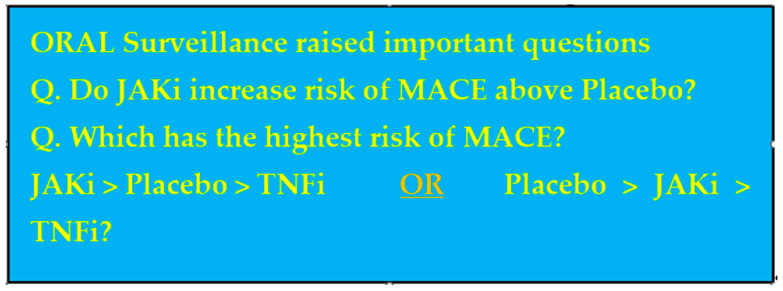
ORAL Surveillance’s MACE Uncertainty [36].

The interpretation of these results may not be as straightforward as they initially appear, however. It is understood and appreciated that RA disease activity is associated with an increased risk of MACE [37]. RA patients have an approximately 70% higher risk of cardiovascular disease compared with the background general population [38]. It has been well established that a reduction in inflammation and disease activity in RA with the use of traditional DMARDs such as MTX and biologics such as TNF inhibitors significantly reduce the risk of MACE [39,40]. Given the undoubted beneficial effects of JAK inhibitors on swollen joint count, pain scores and other markers of RA disease activity, it is initially surprising that such a potent reduction in systemic inflammation in this class is associated with a higher risk of MACE. The mechanism of action that explains this remains unclear. Perhaps we cannot see the wood for the trees! It may well be that the perceived higher risk of MACE observed between TOFA and TNFis in ORAL Surveillance can be partly attributed to a greater reduction in MACE from the baseline with TNFis. As such, ORAL Surveillance raises this important question highlighted in Figure 3: is the risk of MACE highest with JAKi treatment or placebo? Unfortunately, there was no placebo comparator group to help determine if TOFA increases the risk of MACE from the baseline or decreases the risk from the baseline. In any case, it is apparent that TNFis fare more favourably than TOFA in terms of risk of MACE in an older group of RA patients with significant cardiovascular risk factors. As a result, the TNFi class should remain the first line biologic class in the treatment of RA in elderly patients.

Since ORAL Surveillance, long term safety data from RA patients of all ages treated with TOFA, along with safety data from the trials of other JAKis, suggests that JAKi treatment results in MACE incident rates in line with the healthy general population without RA, at an incident rate of 0.4 to 0.6 per 100 patient years [41,42,43]. This is somewhat reassuring, suggesting that the JAKi class still reduces cardiovascular risk compared to no treatment, albeit that the TNFis’ beneficial effect on cardiovascular risk may yet be more potent.

Further reassuring evidence comes from the STAR-RA study from the US which included >102,000 RA patients treated with either TOFA (*n* = 12,852) or a TNFi between 2012 and 2020. TOFA and TNFi treated patients had a similar risk for the primary composite outcome of hospitalisation for myocardial infarction (MI) or cerebrovascular accident (CVA), with a non-significant hazard ratio (HR) of 1.01 after adjustment for confounders. Similarly, the HRs for the secondary outcomes of MI, CVA, heart failure and coronary artery revascularisation were non-significant ranging from 0.93 to 1.07. Lastly, when attempting to replicate ORAL Surveillance’s over 65 group with cardiovascular risk factors, the sub analysis demonstrated that TOFA treated patients had a numerically higher risk for the primary composite compared to the TNFi treated patients, but this trend did not reach statistical significance with a HR of 1.24 [44].

## 8. Thromboembolism

In addition to cardiovascular risk concerns with the JAKi class, there are also concerning signals for increased venous thromboembolism risk (VTE) with both TOFA and BARI [45]. The FDA mandated ORAL Surveillance trial revealed an increase in the risk of pulmonary embolism (PE) and death with a 10 mg twice daily dose of TOFA in RA patients when compared with a TNFi. This dose is not approved for RA and is only approved for ulcerative colitis. Discovery of this increased risk of PE over time contrasts with the initial reassuring data derived from pooled analyses of RCTs [36,46]. Even prior to ORAL Surveillance, concerning safety signals appeared during the trials of Eli Lilly’s Olumiant (Baricitinib) regarding increased risk of venous thromboembolism (VTE), suggesting a possible class effect of JAK inhibitors, rather than a risk associated solely with tofacitinib and strong JAK1/JAK3 inhibition [8]. Quite surprisingly, there was a definite increased risk of VTE with the baricitinib 4 mg group in the first 12 weeks of treatment. However, this increased risk dropped off thereafter, with both the 2 mg and 4 mg baricitinib doses displaying long term incidence rates of VTE of 0.5 events per 100 patient years which is in line with population-based RA cohort studies and registry data [8,47].

To provide some context, much like cardiovascular disease in RA, it has been established that RA patients are also at a higher risk of VTE than the healthy background population. A nationwide registry-based cohort study from Sweden found that RA patients were 1.88 times more likely to develop VTE than healthy controls [48]. The consensus opinion is that tight disease control should reduce VTE risk. Again, given the potent effect of the JAKi class on systemic inflammation, intuition would lead us to believe that this class of therapeutic drugs would be associated with a lower risk of VTE than other classes of bDMARD, but the early data from clinical trials of TOFA and BARI and ORAL Surveillance would suggest the opposite. The reality can be confusing and counter intuitive. For example, a large real-world cohort of elderly RA patients from the US showed that MTX treatment was associated with a twofold increased risk of VTE relative to hydroxychloroquine (HCQ) [49]. The reality of this result is hard to reconcile with the first principles idea of “better disease control, lower VTE risk”. No one is going to argue that HCQ is a more potent DMARD than MTX so perhaps the answer lies in channelling bias. In general, those with milder disease are easily controlled on HCQ whereas those with more severe refractory disease tend to be treated with MTX. Of course, there may be some anti-thrombotic quality to HCQ itself.

In contrast to the FDA black box warnings, a meta-analysis from 42 RCTs did not find an increased risk for VTE in RA patients on JAKis compared to those on a placebo [50]. As mentioned, RA itself confers an increased risk of VTE, particularly if the disease is active. This meta-analysis may ease some of the concern that the JAKi class is intrinsically pro-thrombotic. Again, similar to MACE concerns, ORAL Surveillance would suggest that perhaps TNFis are just better at reducing VTE risk than JAKis.

Real-world evidence from Swedish registry data for >85,000 patients with RA on a JAKi or TNFi did not show any increased VTE risk with TOFA [48]. These findings were also consistent with data from the US CORRONA RA registry which also showed similar rates of VTE among 1999 RA patients on TOFA and 8358 on other bDMARDs [51].

Lastly, the pandemic did provide the opportunity to learn a little more about the JAKi class. BARI 4 mg OD given for 2 weeks as a COVID treatment did not increase the risk of VTE in an illness with an inherently increased risk of thrombophilia [52]. While all of this is reassuring, JAKis are still best avoided in elderly patients with risk factors for VTE.

## 9. Malignancy

Along with MACE and VTE, the chronic systemic inflammatory state of uncontrolled RA increases the overall risk of malignancy compared with the general population [53]. Intuition would tell the clinician that controlling systemic inflammation should reduce the risk of malignancy. At the same time there is an appreciation that long term immunosuppression in transplant patients and previous chemotherapy in oncology patients can increase the risk of malignancy at a later point in life. It stands to reason then that there is a “goldilocks zone” of immunosuppression in RA. There remains the concern that the JAKi class may have overstepped this mark due to the reduced circulating levels of interferon and natural killer cells seen with treatment [54,55]. Is the risk of cancerous cells evading immunosurveillance real?

In the ORAL Surveillance study, during a median follow-up period of 4 years, TOFA did not meet the non-inferiority criteria, with an increased HR of 1.48 for cancers [36]. Interestingly, this disparity between TOFA and TNFis was skewed by patients enrolled in the North American study centres, were there was an increase in the rate of lung cancers and lymphomas, particularly amongst the older smokers enrolled.

More recently, the King’s College Group performed an extensive meta-analysis examining the association of the JAKi class with malignancy when compared to placebo, TNFi class and MTX [6]. This comprehensive meta-analysis identified 78 clinical trials for inclusion, 62 randomised control trials (RCT) and 16 long term extensions (LTE) trials of inflammatory joint, skin and bowel diseases, treated with JAKis. JAKis were not associated with a higher incidence of malignancy compared with the placebo or MTX. JAKis were however associated with a higher incidence of malignancy compared to TNF inhibitors.

The authors note that this observation was primarily due to the ORAL Surveillance trial. If ORAL Surveillance is excluded from this analysis, there is a trend towards a higher incidence of malignancy with JAKis compared to TNFis, but this difference is no longer statistically significant. The authors conclude that cancer events are rare with all treatments, with an overall incidence rate of one event per 100 person-years of exposure.

## 10. Infections

As with most bDMARDs, the most commonly reported adverse events are infections [56]. Undoubtedly the incidence of common infections such as upper respiratory tract infections, lower respiratory tract infections and urinary tract infections is certainly higher than the healthy background population not on immunosuppression. The incidence of infections in RA patients on JAKis is similar to those on bDMARDs, however [56,57]. ORAL Surveillance tells a somewhat different story, with higher rates of infections seen with TOFA at either dose compared with TNFi. While non-serious infections (NSI) are increased with TOFA 5 mg BD and TOFA 10 mg BD exposure compared with TNFis, there was only a statistically significant difference between TOFA and the TNFis in terms of serious infections events (SIE) at the higher 10 mg BD dose. The biggest determinants or risk factors for SIE were increasing age, history of chronic lung disease and opioid use for the TOFA 5 mg BD dose, and increasing age for the TOFA 10 mg BD dose. At the current licensed dose of 5 mg BD for RA, the number needed to harm (NNH) versus TNFis to produce one additional SIE is about 48 over a 5 year period, or approximately 238 patients in one year [58].

With regard to the FDA approved JAKis (TOFA, BARI and UPA), meta-analyses have demonstrated similar rates of infections with each drug [8,41,42,59]. The main risk factors for infection are, unsurprisingly, advanced age, glucocorticoid use (prednisolone ≥ 7.5 mg/day), disease activity, diabetes and higher dose (i.e., TOFA 10 mg BD which is no longer licensed for RA) [46,60,61]. Interestingly, real word data from the German RABBIT registry showed that even in elderly patients, JAKis, bDMARDs and csDMARDs were all associated with similar rates of infections [61]. This in contrast to the findings of ORAL Surveillance.

## 11. Herpes Zoster Infection

Herpes Zoster (HZV) infection is seen more frequently in RA patients than in the general population. A combination of advancing age and immunosuppression often due to chronic glucocorticoid exposure increases risk of HZV infection [62]. Pooling safety data from the TOFA RCTs revealed an incidence rate of HZV infection of 1.5 to 2-fold higher than normally seen in the RA population treated with csDMARDs and bDMARDs [1]. This finding is corroborated by real world data from the American CorEvitas register, where the HR for HZV infection was 2.32 (95% CI 1.43–3.75) for TOFA compared with bDMARDs [51].

Noteworthy is the non-uniform geographic distribution of HZV risk with the JAKi class. HZV infection rates were as high as 9.2 per 100 patient-years in Asia and 8.9 per 100 patient-years in India. HZV infection rates with JAKi treatment were much lower in North America and Europe at 3.3 and 2.7 per 100 patient-years, respectively. Differences in childhood exposure to HZV or HZV vaccination programmes in rheumatology patients in the East versus the West, or alternatively genetic factors, may influence this geographic distribution of HZV infection in RA patients. It has been postulated that JAKi downregulate interferon and interleukin-15 (IL-15) to such a degree that it tangibly effects viral elimination and thus increases risk of HZV infection. Fortunately, severe multi-dermatomal and disseminated HZ is rare [63,64].

In studies of TOFA, the most significant risk factors for HZV infection were increasing age, glucocorticoid use, combination treatment with background MTX, smoking and higher JAKi dose [65]. Lastly, whether or not JAK-1 selectivity is associated with a lower risk of HZV infection still needs clarification.

## 12. Conclusions

There is certainly a role for JAKis in younger patients without significant cardiovascular risk and with active disease refractory to multiple classes of bDMARD. However, it is hard to justify their use as a first-line advanced therapy in the majority of patients, particularly given the greater wealth of safety data about TNFis, tocilizumab and abatacept in real world practice. Additionally, in many countries the JAKi class will remain a more expensive therapeutic option as it will be some time before generics arrive to market.

**Figure 4:**
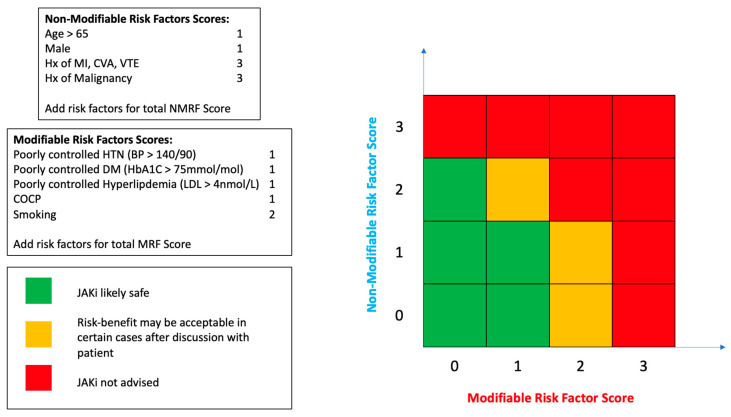
A Tool for Approximate Risk Stratification in JAKi Prescribing.

For now, the wait goes on for more long-term surveillance studies that may ease our concerns with the JAKi class. The FDA mandated-post marketing safety study for BARI will not finish until 2026 (NCT03915964) and AbbVie’s SELECT-COMPARE 5-year extension phase safety data for UPA is still awaited. It is prudent to continue to use JAKis judiciously on a case-by-case basis until the real-world picture becomes clearer. To this end, we present a simple prescribing guidance tool in Figure 4 which aims to risk-stratify patients. Today the practising rheumatologist has many therapeutic options at their disposal, and the speciality is no longer in an era where it is desperate for any efficacious drug regardless of adverse events. Given the impact the TNFi class has had over the past quarter of a century, rheumatology is now at a point in RA therapeutic discovery that more is expected of a new drug than ever before. While TNFis are still the first port of call in terms of bDMARDs, JAKis may be used more frequently in the future in younger patients, for refractory disease and in those who prefer to avoid self-injection. This, however, must be a shared informed decision based on the known adverse event profile.

## 13. Limitations

This review article focuses on the three FDA approved JAKi in RA; TOFA, BARI and UPA. The efficacy and safety of peficitinib and filgotinib are not reviewed or discussed in detail. Safety and post-marketing data from TOFA should not necessarily be applied to other JAKi with different selectivity. Caution is therefore advised in extrapolating and applying such results to other JAKi.

## Data Availability

This narrative review article contains no newly created data.
